# The salt secretion of leaves promotes the competitiveness of Reaumuria soongarica in a desert grassland

**DOI:** 10.1186/s12870-022-03457-4

**Published:** 2022-02-25

**Authors:** Chang-shun Wang, Hui-qing Wang, Wei Wang, Cun-zhu Liang, Hua-min Liu, Li-xin Wang

**Affiliations:** 1 School of Ecology and Environment, Inner Mongolia University, Hohhot, 010021 Inner Mongolia China; 2 Scientific Research Department, Hulunbeir College, Hulunbeir, 021008 Inner Mongolia China; 3 Hulunbeir Meteorological Bureau, Hulunbeir, 021000 Inner Mongolia China

**Keywords:** Stress tolerance, Interspecies competition, Tradeoff, Soil conductivity, Inner Mongolia

## Abstract

**Background:**

For better understanding the mechanism of *Reaumuria soongarica* community formation in a salt stressed grassland ecosystem, we designed a field experiment to test how leaves salt secretion changes the competitive relationship between species in this plant communities.

**Results:**

Among the three species (*R. soongarica, Stipa glareosa* and *Allium polyrhizum*) of the salt stressed grassland ecosystem, the conductivity of *R. soongarica* rhizosphere soil was the highest in five soil layers (0–55 cm depth). The high soil conductivity can increase the daily salt secretion rate of plant leaves of *R. soongarica*. In addition, we found the canopy size of *R. soongarica* was positively related to the distance from *S. glareosa* or *A. polyrhizum*. The salt-tolerance of *R. soongarica* was significantly higher than the other two herbs (*S. glareosa* and *A. polyrhizum*). Moreover, there was a threshold (600 µS/cm) for interspecific competition of plants mediated by soil conductivity. When the soil conductivity was lower than 600 µS/cm, the relative biomass of *R. soongarica* increased with the soil conductivity increase.

**Conclusions:**

The efficient salt secretion ability of leaves increases soil conductivity under the canopy. This leads the formation of a “saline island” of *R. soongarica.* Meanwhile *R. soongarica* have stronger salt tolerance than *S. glareosa* and *A. polyrhizum.* These promote the competitiveness of *R. soongarica* and inhibit interspecies competition advantage of the other two herbs (*S. glareosa* and *A. polyrhizum*) in the plant community. It is beneficial for *R. soongarica* to establish dominant communities in saline regions of desert grassland.

**Supplementary Information:**

The online version contains supplementary material available at 10.1186/s12870-022-03457-4.

## Background

There are two sides of the same coin between species establishment and community stability [1–3]. The zonal plant communities alternate along environmental gradients [4] and have clear and stable boundaries [5, 6]. However, the typical highly drought-tolerant desert plant *Reaumuria soongarica* has a unique distribution [7]. *R. soongarica* not only dominates desert communities in Asia, but also forms communities in the eastern Mongolian Plateau grassland where soils are generally saline [8]. Therefore, *R. soongarica* has managed not only adapt to the extreme drought of the desert environment but also occupy areas with high moisture, where other plants dominate with different strategies across vegetation zones [7]. Generally, a zonally dominant species cannot spread to other vegetation zones [9]. This raises the question that how *R. soongarica* breaks this general rule?

In reality, some plant species cannot adapt to environmental stress, whereas others that have adapt to a stressed environment cannot spread to areas with greater precipitation or higher temperature regimes [10]. For example, the desert steppe prevents the establishment of desert plant seedlings via strong interspecific competition imposed by its dominant populations, which maintains the relative stability of the vegetation distribution [11, 12]. Conversely, steppe plants have difficulty adapting to harsh desert habitats and cannot effectively survive in the desert [13, 14]. This phenomenon is the result of the combined effects of plant physiological tolerance to abiotic stress and interspecies competition (i.e., the ability of a plant to inhibit the growth of another plant) [15]. It remains unclear how the environmental tolerance and interspecies competition of plants act on establishment of *R. soongarica* in saline regions within the steppe.

*R. soongarica* is able to secreted Na^+^ through salt gland to alleviate the toxic effects of Na^+^ and maintain water status in plant, which contribute to stimulate the growth of plant [16]. The secrete salt from the leaves of *R. soongarica* deposit on the ground [17]. This is similar to *Mesembryanthemum crystallinum* that can absorb salt from deeper soil layers and deposit the salt on the soil surface, and lead to the death of neighboring, less salt-tolerant plants, and ultimately form a dominant community [18]. Owing to the limited tolerance of a given plant to its environment, this interspecies competition advantage is not identical across environments [19, 20].

The tolerance of plants to extreme environments are based on specialized functions [21, 22]. In a stress-less environment, these special functions are redundant and may become a burden on interspecific competition [23]. Thus, plants with higher tolerance will show a reduced ability in terms of access to resources under interspecies competition in a stress-free environment [3, 24]. We assumed that *R. soongarica* was unable to occupy areas with better environmental conditions (i.e., vast grassland areas, not just salty areas) because it cannot compete with local dominant species, especially herbs.

Our study aimed to answer this question by focusing on the adaptability of *R. soongarica* to stress and interspecies competition in a grassland community. Therefore, the first hypothesis of the present study is that whether the soil salinity under *R. soongarica* is higher than that of herbs, and whether the presence of *R. soongarica* affects the position of the herb. The second hypothesis of the present study is that *R. soongarica* is unable to compete with dominant plants in grassland areas under conditions of low soil salinity.

## Results

### Soil conductivity and the plant community distribution

**Fig. 1:**
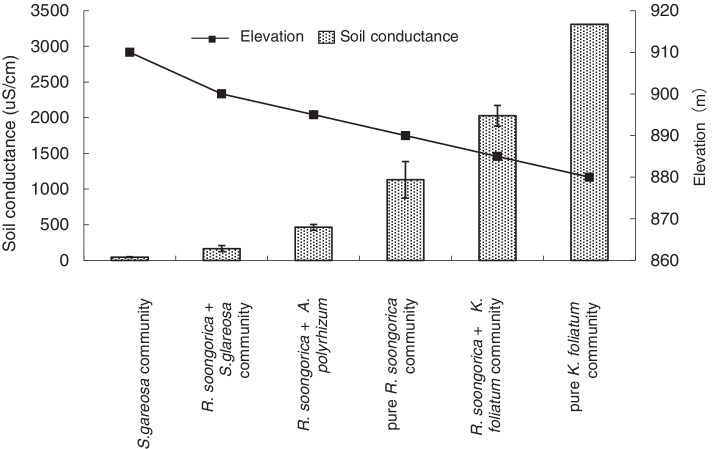
Mean soil conductivity (0–55 cm) and elevation in different communities at the research site. The vertical lines at the top of the bars represent the standard deviation (*N* = 3)

The soil conductivity (soil salinity) increases with the decrease of elevation, which produces clear boundaries between the plant communities in the basin (Fig. 1). Outside the ancient lake basin is the zonal plant community, which is dominated by *Stipa glareosa*. From the basin margin to the center, the plant communities are *R. soongarica* + *S. glareosa*, *R. soongarica* + *Allium polyrhizum*, pure *R. soongarica*, *R. soongarica* + *Kalidium foliatum*, and pure *K. foliatum*.

### Rhizosphere soil conductivity of the different plant species

**Fig. 2:**
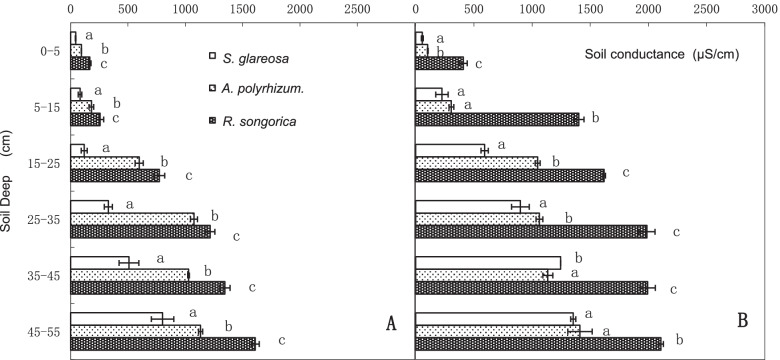
Soil salt conductivity under the three species (*Reaumuria soongarica*, *Stipa glareosa*, and *Allium polyrhizum*) in the *R. soongarica* + *S. glareosa* community (**A**) and the *R. soongarica* + *A. polyrhizum* community (**B**) at different soil depths (mean ± standard error; *N* = 9). Lowerercase letters indicate significant differences among the three species at different soil depths according to Duncan’s test (*p* < 0.05)

The rhizosphere soil conductivity of *R. soongarica*, *S. glareosa*, and *A. polyrhizum* increased with the increase of soil depth (Fig. 2). *R. soongarica* had significantly higher rhizosphere soil conductivity than *S. glareosa* and *A. polyrhizum* in all soil depths (*P* ≤ 0.001) (Fig. 2). In *R. soongarica and S. glareosa* community, *S. glareosa* showed the lowest rhizosphere soil conductivity (Fig. 2A)*.*

### Salt secretion rate of *R. soongarica*

**Fig. 3:**
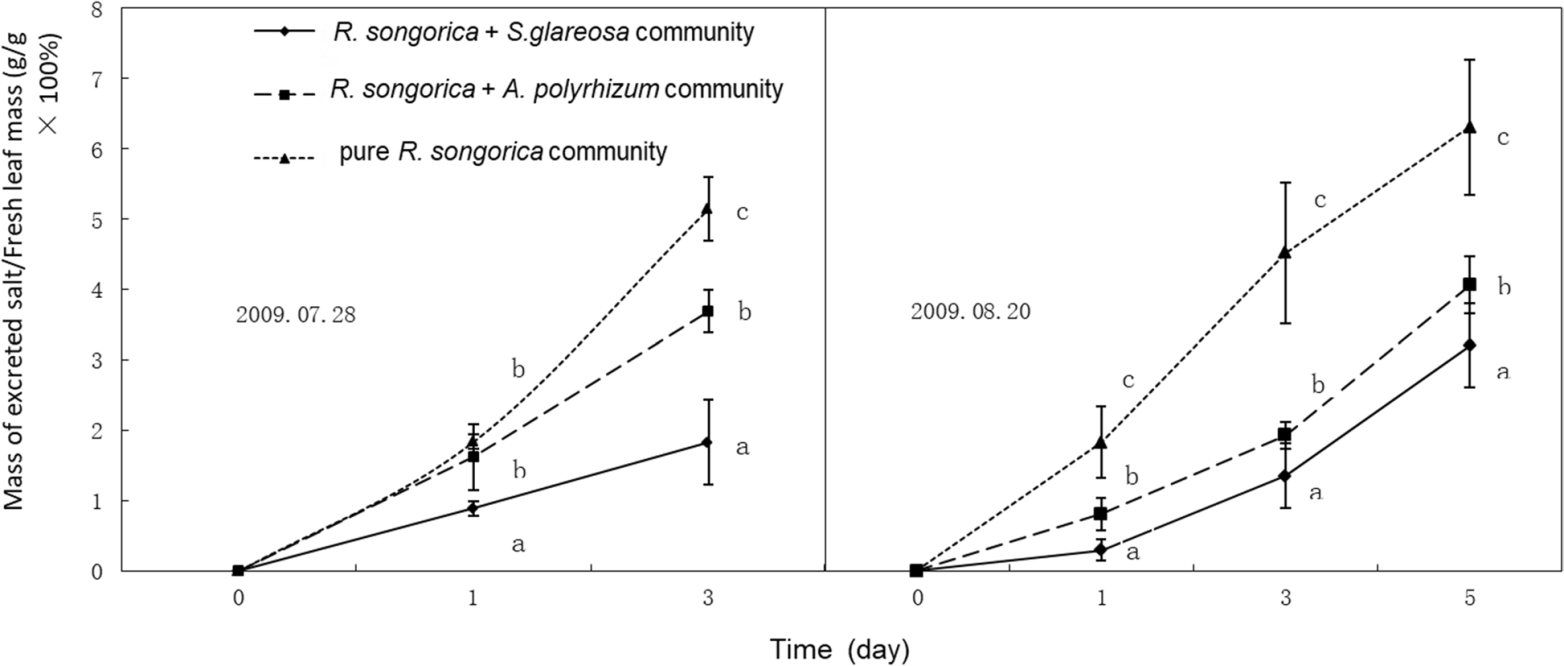
Quantitative analysis of salt excreted by *R. soongarica* in different communities (*R. soongarica* + *S. glareosa*, *R. soongarica* + *A. polyrhizum,* and pure *R. soongarica*) with different soil conductivities at two different times (mean ± standard error; *N* = 9). Lowercase letters indicate significant differences among the communities on a different day according to Duncan’s test (*p* < 0.05)

There were significant differences in leaf salt secretion rates among the three communities (*P* < 0.01). The salt secretion rate increased with the increase of soil conductivity (Fig. 3). The daily salt secretion rate ranged from 1 to 2% of the fresh leaf weight in different communities. The amount of salt excreted increased over time.

### Population distribution patterns in the community

**Fig. 4:**
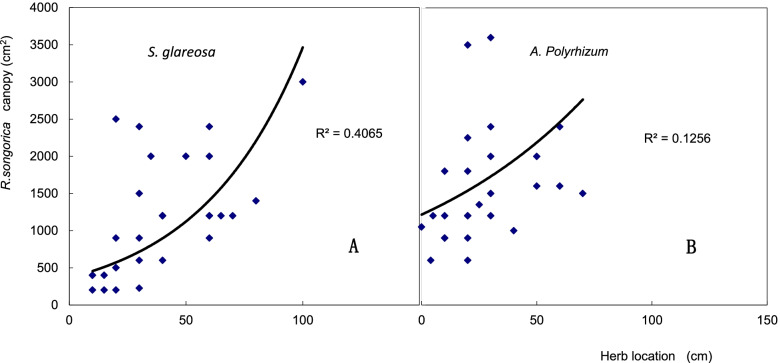
Correlation between *Reaumuria soongarica* canopy size and its closest distance to *Stipa glareosa* (**A**) and *Allium polyrhizum* (**B**)

With an increase of the canopy size of *R. soongarica*, *S. glareosa* and *A. polyrhizum* were situated further away from the shrub (Fig. 4). The correlation between the *R. soongarica* canopy diameter and the distance to the nearest *S. glareosa* (*R*^2^ = 0.4065; *P* < 0.05) was higher than that to the nearest *A. polyrhizum* (*R*^2^ = 0.1256; *P* < 0.05).

### Effect of salinity on plant growth

**Fig. 5:**
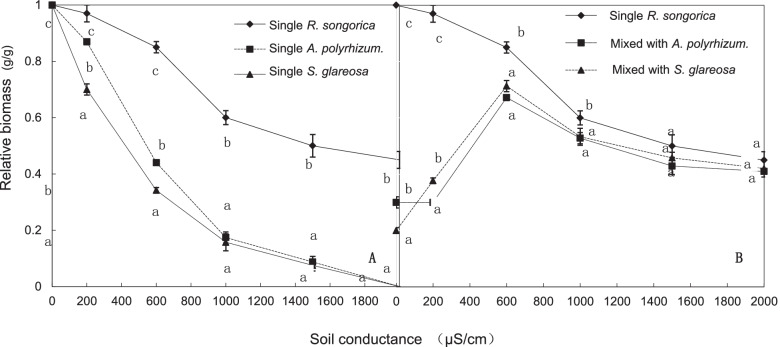
Relative biomass of the three species (*Reaumuria soongarica*, *Stipa glareosa*, and *Allium polyrhizum*) cultivated separately (**A**), and of *R. soongarica* cultivated individually or with herbs (**B**). Values are means ± standard error (*N* = 3). Lowercase letters indicate significant differences among different conditions according to Duncan’s test (*p* < 0.05)

Soil conductivity had a negative effect on plant growth (Fig. 5A). The plant relative biomass decreased with an increase of the soil conductivity, and the relative biomass of *R. soongarica* decreased more slowly than *S. glareosa* and *A. polyrhizum* (*P* < 0.001, *N* = 54). The relative biomass of the two herbs at 600 µS/cm was decreased to less than 50%, whereas that of *R. soongarica* was decreased to 80%. The relative biomass of the shrub was decreased to 50% at 2000 µS/cm, whereas that of the two herbs fell almost to zero under the same conditions. At low soil conductivity (200 or 600 µS/cm), the relative biomass of *S. glareosa* decreased slightly faster than that of *A. polyrhizum*; therefore, *A. polyrhizum* was more salt-tolerant than *S. glareosa.*

### Effect of interspecies competition on the growth of *R. soongarica*

The relative biomass of *R. soongarica* decreased significantly when planted with *S. glareosa* or *A. polyrhizum* at low soil salinity (soil conductivity below 600 µS/cm; *P* < 0.001; Fig. 5B). However, there was no significant difference in relative biomass between single planting *R. soongarica* and mixed planting at high soil salinity (soil conductivity over 1000 µS/cm; *P* = 0.1). The herbs significantly limited the growth of *R. soongarica* seedlings at low soil conductivity (≤ 600 µS/cm); however, their effect was eliminated at higher soil conductivity (≥ 1000 µS/cm), which was detrimental to the herbs.

## Discussion

### Competitiveness and environment

Processes related to special functions can alter the local environment. Thus, the limited competitiveness of the original vegetation [25–27] is important for the successful establishment of plant species in the community [2, 3]. Our results showed that the conductivity of every soil layer was significantly higher for *R. soongarica* than for the two herbs (Fig. 2) and the higher the soil conductivity, the higher the salt secretion of *R. soongarica* (Fig. 1 and 3). Therefore, the physiological process of salt secretion from *R. soongarica* leaves created the spatial heterogeneity of soil salt in the coexisting community, which was important for its establishment in the grassland.

A plant can inhibit the growth of its competitors by developing special physiological functions, thus to enhance its ability to obtain resources and improve species competitiveness [28, 29]. In our study, *R. soongarica* rhizosphere soil had high electrical conductivity, forming a “saline island”. This may be due to the strong salt secretion ability of *R. soongarica* leaves (Fig. 3). Moreover, the increase of *R. soongarica* canopy can promote the growth of saline island, and drive away plant species with low salt tolerance. This may be the reason why *S. glareosa* and *A. polyrhizum* are planted far away from *R. soongarica* (Fig. 4). Our results support first the hypothesis that salt secreted by *R. soongarica* leaves can change the soil salinity and inhibit the growth of plant species with low salt tolerance, thereby to change the structure of plant community.

Plants can gain higher competitiveness through allelopathy [30], which increases their chances of establishment [23]. This phenomenon is not always the result of a specialized physiological behavior. For example, oak leaves can inhibit the growth of herbaceous plants [31] and the removal of alpine forest litter can considerably promote the growth of herbs [32]. Thus, the ability to change the environment (i.e., allelopathy) is key point for the survival of plants in this community; it changes the level of interspecies competition and ultimately leads to changes in the community structure.

### Competitiveness and tolerance

Many drought- or salt-tolerant species grow better in a stress-free environment [25, 33]; however, the realized niche of most stress-tolerant plants often deviates from their fundamental niche because of interspecies competition [15]. The results of our pot experiment showed that the relative biomass of *R. soongarica*, *S. glareosa* and *A. polyrhizum* decreased in the soil environment with high conductivity. In other words, these three plant species did not depend on salt physiologically, and preferred the environment without salt stress (Fig. 5A). Additionally, the effect of soil salinity on the growth of the two herbs was much greater than that of *R. soongarica*. That is, *R. soongarica* had better tolerance to high salinity environment.

In our pot experiment, the competitiveness of *R. soongarica* is not strong enough, and the growth of its seedlings is inhibited by *S. glareosa* and *A. polyrhizum* in the low salinity soil environment (Fig. 5B). On the desert steppe in eastern Inner Mongolia, the soil salinity is not extremely high. The shrub species (*R. soongarica*) with more salt tolerance had no interspecific competitive advantage with herbs (*S. glareosa* and *A. polyrhizum*), and could not establish a wide range of dominant communities [7]. Although *R. soongarica* can adapt to drought and salinity, it has no interspecific competitive advantage in low salinity soil environment. A species with both high stress resistance and interspecies competitiveness will reduce species diversity in communities. However, this is not consistent with the result of long-term evolution. In fact, plants may tradeoff between different adaptive abilities (e.g., tolerance and interspecies competitiveness, or acquisitive and conservative) [34, 35]. Many studies have also confirmed the exclusive relationship between environmental tolerance and resources competitiveness [25]. At low soil nutrient levels, species with high tolerance are more successful. Conversely, when the soil nutrient level is high, the same species are at a competitive disadvantage [36]. Environmental pressure limits the competitiveness of species with strong resource acquisition ability, which in turn increases the competitiveness of highly stress resistant species [24]. *R. soongarica* has evolved salt glands to adapt to drought in desert regions or high-salinity grasslands. However, this specialized trait and capability provides no advantage in non-specific environment. And the plant becomes powerless when competing with dominant grassland species without these specializations [37].

Successful species establishment is easy to understand based on the assumption that the competitiveness of the species distributed across zones is greater than that of the native species [3, 27]. However, the long-term presence of the vegetation zone proves that this assumption is not universally true [35]. Our results support another view that competitiveness is not “real,” but a process related to special functions [28]. The ecological mechanisms of *R. soongarica* establishment, which rely on salt secretion, facilitate the spread of *R. soongarica* across vegetation zones and the formation of a community on the desert steppe in eastern Inner Mongolia. The soil conductivity of the saline islands (Fig. 2) was high enough to inhibit herb growth (Fig. 5A), and *R. soongarica* grown on a saline island could maintain its growth environment thereby to cope with the competition of herbs (Fig. 5B). Our results add new knowledge to the existing literature, and thus help to advance our understanding of how some shrub species with strong environmental tolerance can survive in desert steppes and establish a stable community. The interspecies competition advantage of a species is inverse to its ability to tolerate abiotic stress, indicating a tradeoff among different abilities, which determines the distribution of plants.

## Conclusion

Our results showed that *R. soongarica* has strong interspecific competitive advantage in high salinity soil environment and can establish a stable dominant community. First, *R. soongarica* can tolerate strong environmental salt stress. Second, the salt secreted by *R. soongarica* leaves can affect soil conductivity and increase soil salinity to form a salt island. The competitive advantage of herb species (*S. glareosa* and *A. polyrhizum*) over *R. soongarica* will be reversed under the condition of high soil salinity. This work is helpful to understand how shrub species with strong environmental tolerance survive and establish stable dominant communities in grassland. Our findings support the hypothesis that the interspecies competition advantage of a species is inverse to its ability to tolerate abiotic stress, indicating a tradeoff among different abilities, which determines the distribution of plants.

## Methods

### Experimental location

Our study site was located in an ancient lake basin in the east of Erlianhot City, Inner Mongolia, China (43°23′16″-43°42′28″ *N*, 112°01′1″-112′01′78″ E; 910 m elevation) (Additional File 1, 2, 3, 4, 5 and 6 ).From 1971 to 2009, mean annual temperature was 4.4 °C with the lowest in January (-17.7 °C) and highest (-23.7 °C) in July, and mean annual precipitation was 137.0 mm (about 67.2% falling in June and August) The diameter of the basin is approximately 15 km and the maximum depth is 30 m. The study area has a grazing history with light intensity, and the most common large herbivores are sheep and camels.

The local government authorized us to collect plant samples. The voucher specimen with the collect number (Zhao 20,080,705) was identified by Professor Liqing Zhao and deposited in Herbarium, Inner Mongolia College (HIMC).

### Experimental design

#### Soil conductivity

In order to test the difference of soil conductivity (soil salinity) in distinct herb and shrub coexisting communities, we collected rhizosphere soil samples from each of three plant species in *R. soongarica* and *S. glareosa* community and *R. soongarica* and *A. polyrhizum* community separately. The sampled soil layers were 0–5 cm, 5–15 cm, 15–25 cm, 25–35 cm, 35–45 cm, and 45–55 cm. Each soil sample had three replicates. A total of 108 soil samples were collected. Soil conductivity was measured using the conductance method (Multi 340i; WTW Xylem Analytics, Weilheim, Germany).

#### Salt secretion rate

To detect the difference of leaves salt secretion rate of *R. soongarica* in the distinct communities, we selected 3 individual *R. soongarica* from three types of plant communities (*R. soongarica* and *S. glareosa*, *R. soongarica* and *A. polyrhizum*, and pure *R. soongarica* community) separately, and each community type has three replicates. One branch per individual was marked randomly. At the beginning of the experiment, the leaf surface of the selected branches was washed repeatedly with distilled water to ensure that the surfaces were salt-free. The salt secretion rate was measured on the first, third, fifth, and seventh day. Each marked branch was soaked in 150 mL distilled water, and used a conductivity meter (Multi 340i; WTW Xylem Analytics) to measure the conductivity of solution. Subsequently, branches were harvested to measure the weight of the fresh leaves using an electronic balance (YP1002N; INESA, Shanghai, China) The experiment was conducted in July 2009 and repeated in August Because herbivore grazing damaged the marked plants, we only obtained two and three complete datasets in July and August, respectively.

#### Population distribution patterns

The amount of salt secretion was found to correlate positively with the crown size of *R. soongarica*. The salt secreted by the *R. soongarica* leaves was likely to increase the soil conductivity, and thus affect the growth of the herbs in the community. This could be demonstrated by the canopy area of *R. soongarica* and the distance between *R. soongarica* plants and the neighboring herbs. To investigate the effect of the salt secretion of the *R. soongarica* leaves on neighbor species, we selected 30 *R. soongarica* randomly, measured their canopy diameter and the distances to nearest *S. glareosa* and *A. polyrhizum* in the *R. soongarica* and *A. polyrhizum* community The crown is assumed to be a perfect circle, and its area is calculated by the diameter.

#### Laboratory cultivation experiment

To verify the changes of competitiveness of different plants under different salt conditions, we conducted a cultivation experiment in laboratory. *R. soongarica*, *S. glareosa,* and *A. polyrhizum* seeds were collected in August 2008, and cultured in March of the next year. First, the seeds were soaked in distilled water for 30 min and treated with 0.1% gibberellin to break the seed dormancy. Then the seeds were placed between double filter papers and germinated in a Petri dish in a dark environment. Finally, the germinated seeds were transplantedinto a paper cup (12 cm height, 7 cm top diameter, and 5 cm bottom diameter) filled with 300 g of river sand (rinsed repeatedly with distilled water to remove any salt and nutrient. Five combinations of three species were prepared: the first was pure *S. glareosa* with six seedlings arranged in a ring at 1 cm from the cup wall, the second was pure *A. polyrhizum*, the third was pure *R. soongarica* with only one seedling in the center of the cup. The other two were to place six *S. glareosa* or *A. polyrhizum* seedlings around one *R. soongarica* respectively (the ratio based on field observation).

Based on local precipitation and soil nutrient levels The seedlings were cultivated in an artificial climate chamber (ZRX-1000 ESW; TESTMART, Hangzhou, China) at 25 °C and 70% relative humidity with a 14 h/10 h light/dark regime. Hoagland nutrient solution (5 mL) was applied every two weeks, and distilled water (10 mL in four times) was added once a week. The cultivation lasted for 12 weeks. In the fifth week, different amounts of NaCl (0, 0.18, 0.54, 0.9, 1.35, and 1.8 g) were added to each cup to simulated six salinity levels (0, 200, 600, 1000, 1500, and 2000 µS/cm soil conductivity). After 12 weeks of cultivation, the entire plant was dried and weighed. The relative biomass of the three species in each treatment was calculated based on the biomass of the plant cultivated individually at 0 µS/cm soil conductivity as a control.

### Statistical analysis

One-way analysis of variance (ANOVA) was used to test the difference of electrical conductivity of rhizosphere soil of three plants species (*R. soongarica, S. glareosa,* and *A. polyrhizum*), the difference of salt secretion rate of *R. soongarica* leaves under different soil salinity levels, and the difference of relative biomass of the three species. Duncan’s multiple comparison was used to evaluate the differences among the treatments. Pearson’s correlation analyses were conducted to examine the correlations between *R. soongarica* canopy size and the spacing between *R. soongarica* and herbs (*S. glareosa* or *A. polyrhizum*) Statistical tests were performed using SPSS version 15.0 for Windows (SPSS Inc., Chicago, IL, USA).

### Material statement

This manuscript complies with institutional, national, or international guidelines and the Convention on the Trade in Endangered Species of Wild Fauna and Flora.

## Supplementary Information

**Additional file 1. **

**Additional file 2. **

**Additional file 3.**

**Additional file 4. **

**Additional file 5.**

**Additional file 6.**

## Data Availability

All data generated or analyzed during this study are included in this published article. *Reaumuria soongarica* is not a rare plant for protection. We have the permission to collect the plant samples by the local government. The voucher specimen with the collect number (Wang 20,080,705) was identified by professor Liqing Wang and deposited in Herbarium, Inner Mongolia College (HIMC).

## Abbreviations

R. soongarica: Reaumuria soongarica
S. glareosa: Stipa glareosa
A. polyrhizum: Allium polyrhizum
K. foliatum: Kalidium foliatum
