# Supplementary figures and images for: The salt secretion of leaves promotes the competitiveness of Reaumuria soongarica in a desert grassland
Source: BMC Plant Biol. 2022 Feb 25;22:85. doi: 10.1186/s12870-022-03457-4 (PMC8876110; doi:10.1186/s12870-022-03457-4)

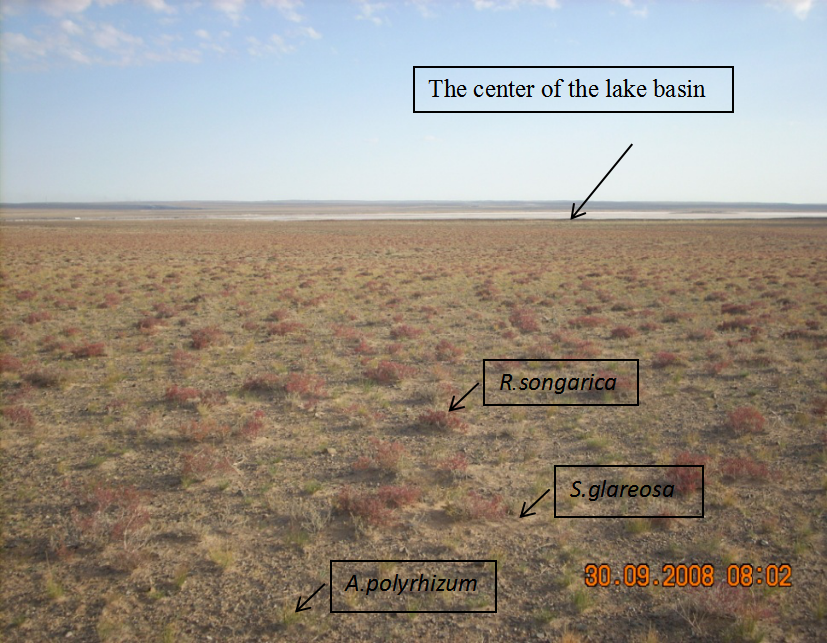

Supplement: Supplementary file 1 — Additional file 1. [file 12870_2022_3457_MOESM1_ESM.png]

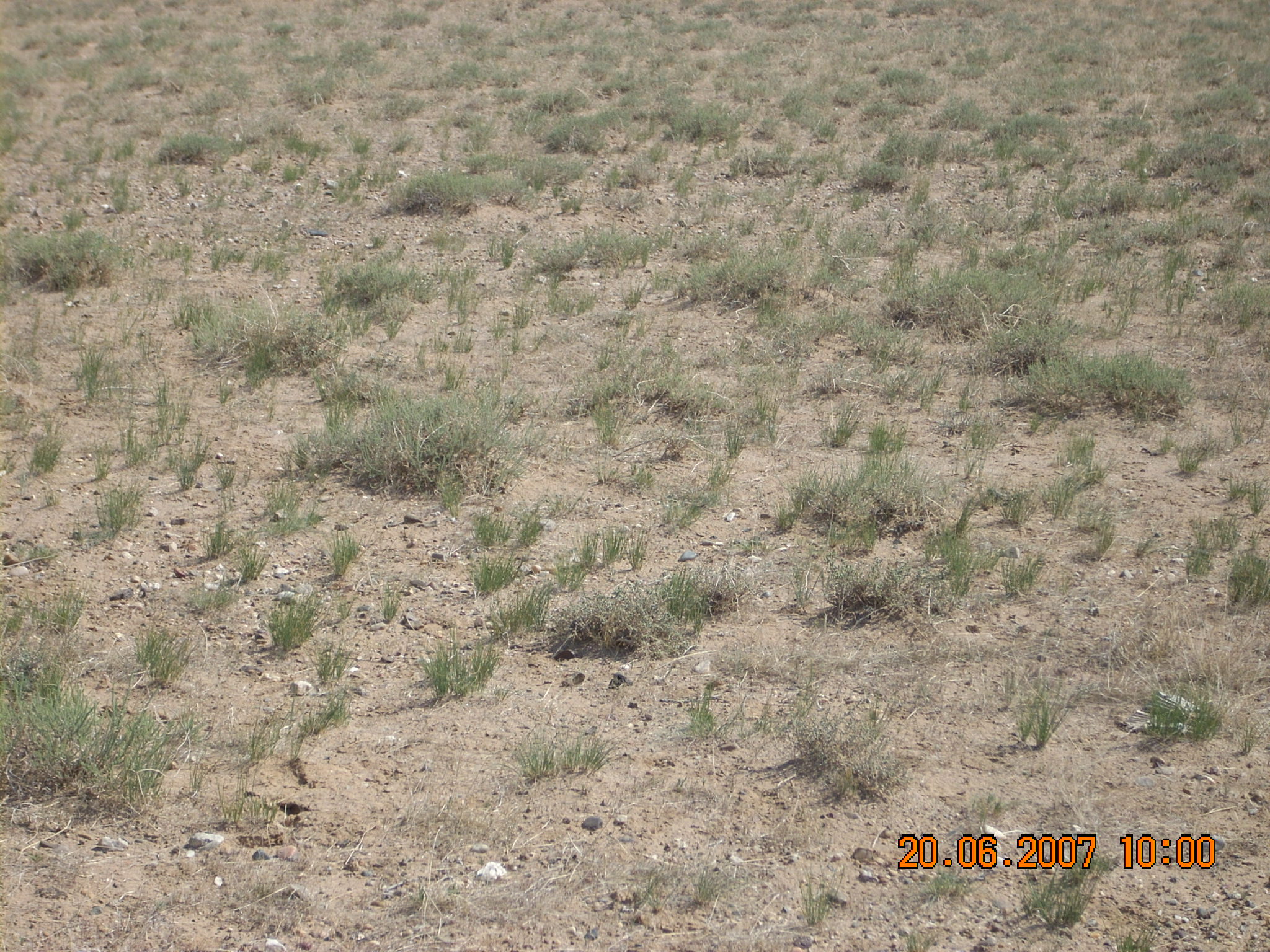

Supplement: Supplementary file 2 — Additional file 2. [file 12870_2022_3457_MOESM2_ESM.jpg]

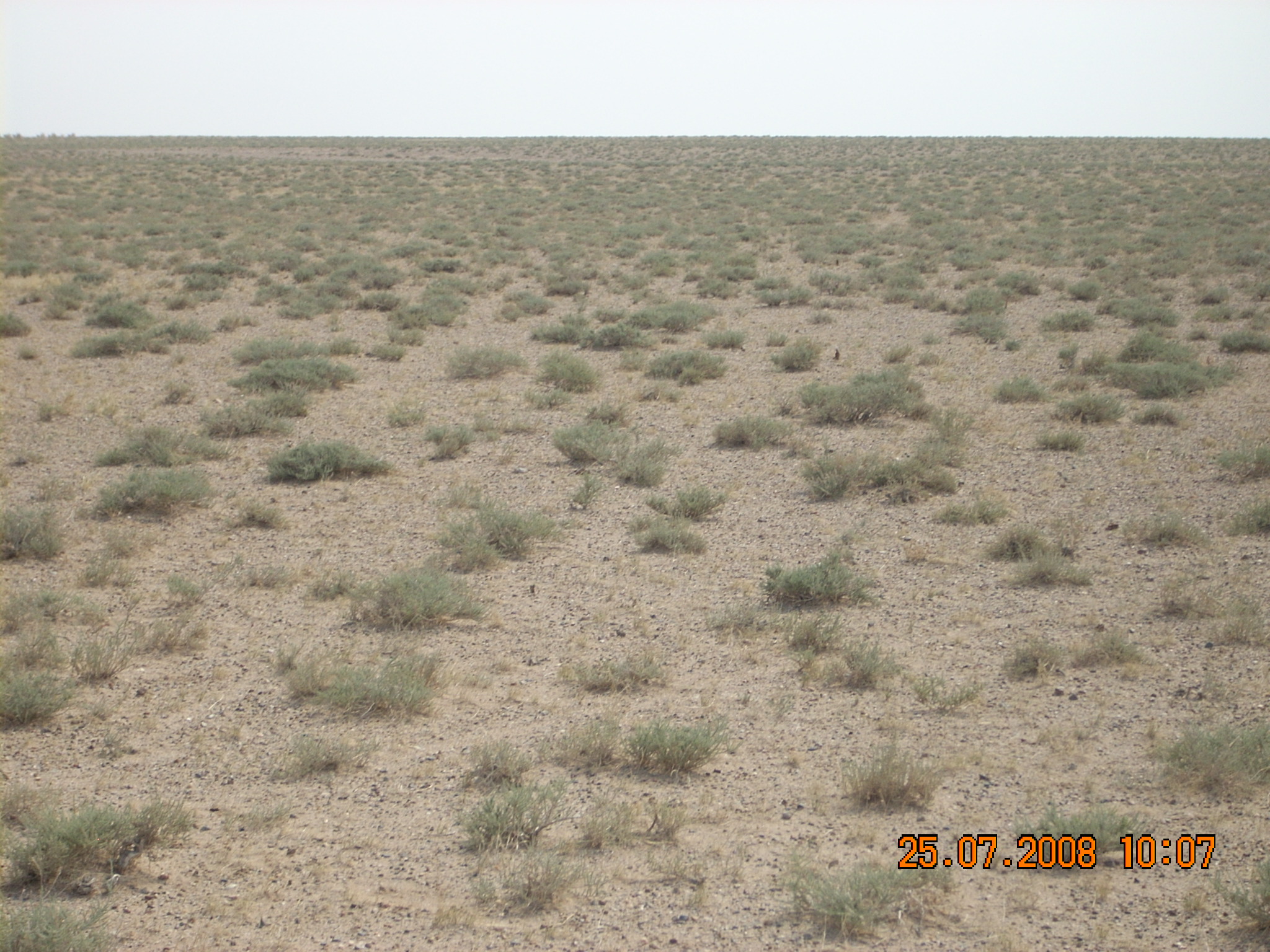

Supplement: Supplementary file 3 — Additional file 3. [file 12870_2022_3457_MOESM3_ESM.jpg]

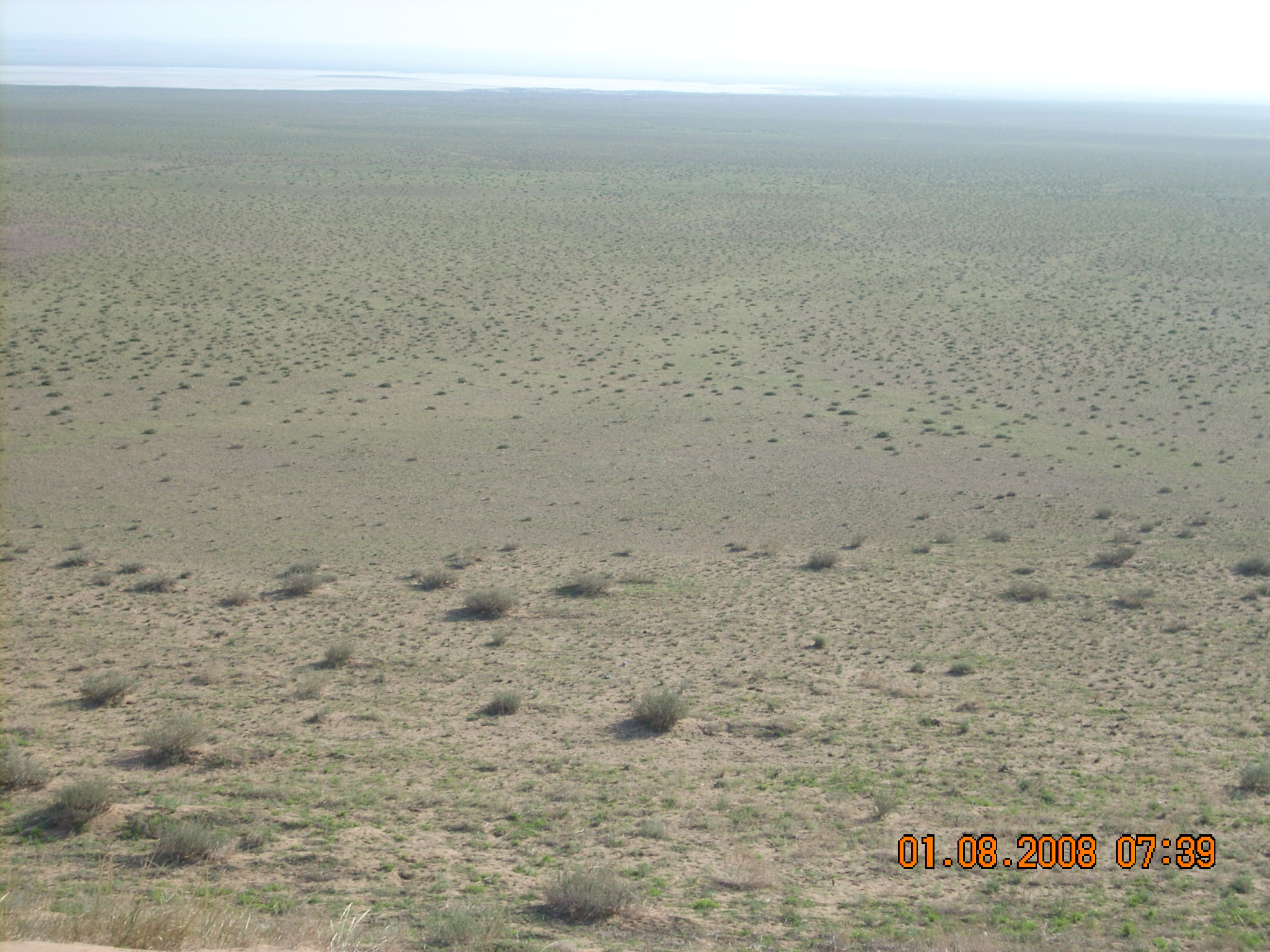

Supplement: Supplementary file 4 — Additional file 4. [file 12870_2022_3457_MOESM4_ESM.jpg]

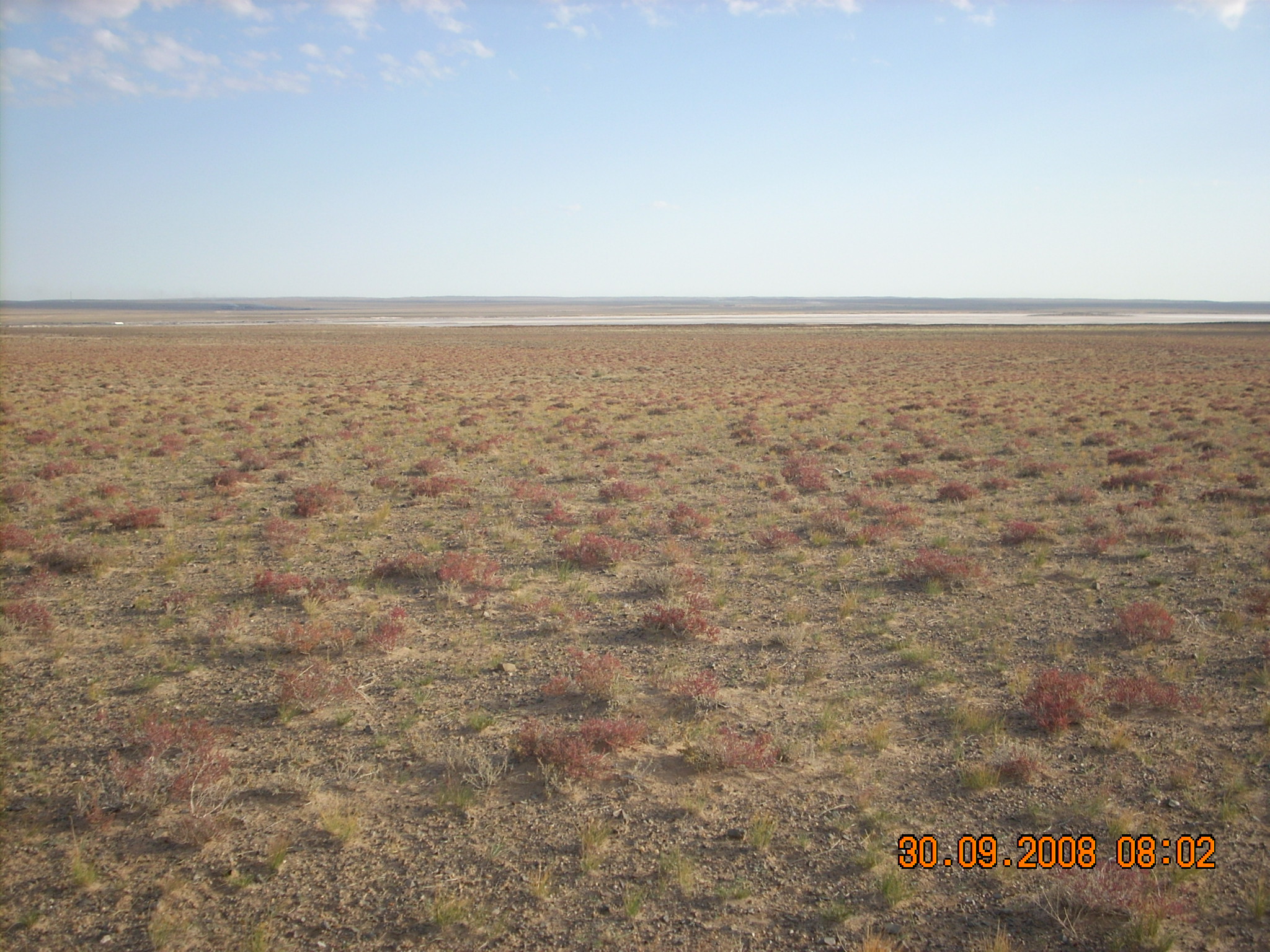

Supplement: Supplementary file 5 — Additional file 5. [file 12870_2022_3457_MOESM5_ESM.jpg]

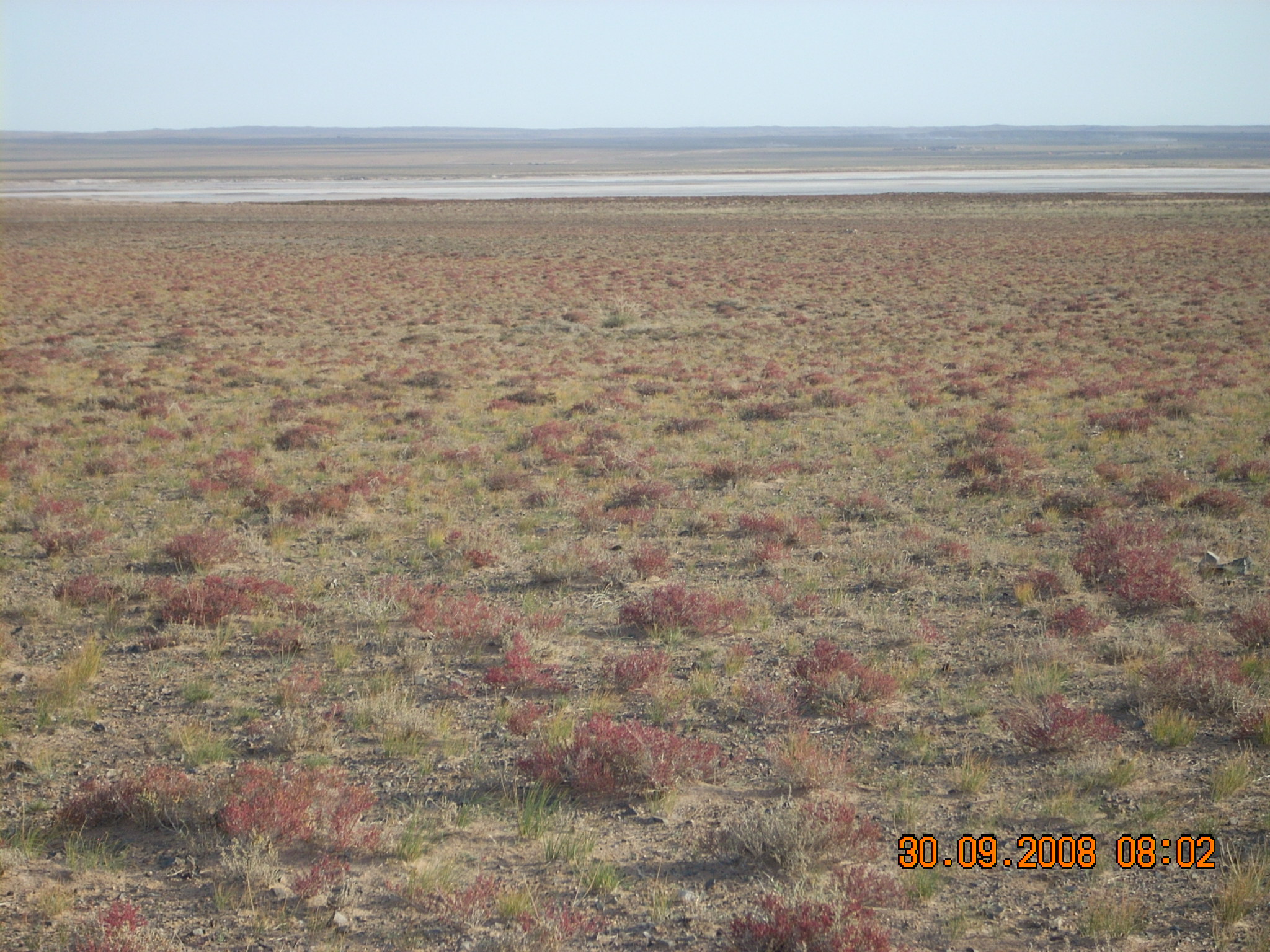

Supplement: Supplementary file 6 — Additional file 6. [file 12870_2022_3457_MOESM6_ESM.jpg]
